# Presence of Antibodies against Sindbis Virus in the Israeli Population: A Nationwide Cross-Sectional Study

**DOI:** 10.3390/v11060542

**Published:** 2019-06-11

**Authors:** Ravit Koren, Ravit Bassal, Tamy Shohat, Daniel Cohen, Orna Mor, Ella Mendelson, Yaniv Lustig

**Affiliations:** 1 Central Virology Laboratory, Ministry of Health, Chaim Sheba Medical center, Tel-Hashomer 52621, Israel; Ravit.Koren@sheba.health.gov.il (R.K.); Orna.Mor@sheba.health.gov.il (O.M.); Ella.Mendelson@sheba.health.gov.il (E.M.); 2 Israel Center for Disease Control, Ministry of Health, Chaim Sheba Medical center, Tel-Hashomer 52621, Israel; Ravit.Bassal@MOH.GOV.IL (R.B.); tamyshohat@gmail.com (T.S.); 3 Department of Epidemiology and Preventive Medicine, School of Public Health, Sackler Faculty of Medicine, Tel-Aviv University, Tel-Aviv 69978, Israel; dancohen@tauex.tau.ac.il

**Keywords:** Sindbis virus, seroprevalence, Sindbis disease, Israel, cross-sectional study

## Abstract

Sindbis virus (SINV) is a mosquito-borne alphavirus circulating globally. SINV outbreaks have been mainly reported in North-European countries. In Israel, SINV was detected in 6.3% of mosquito pools; however, SINV infection in humans has rarely been diagnosed. A serologic survey to detect SINV IgG antibodies was conducted to evaluate the seroprevalence of SINV in the Israeli population. In total, 3145 serum samples collected in 2011–2014, representing all age and population groups in Israel, were assessed using an indirect ELISA assay, and a neutralization assay was performed on all ELISA-positive samples. The prevalence rates of SINV IgG antibodies were calculated. Logistic regressions models were applied to assess the association between demographic characteristics and SINV seropositivity. Overall, 113 (3.6%) and 59 (1.9%) samples were positive for ELISA and neutralization SINV IgG, respectively. Multivariable analysis demonstrated that SINV seropositivity was significantly associated with older age and residence outside metropolitan areas. These results demonstrate that, despite no outbreaks or clinical presentation, SINV infects the human population in Israel. Seropositivity is countrywide, more frequent in people of older age, and less diffuse in Israel’s metropolitan areas. Seroprevalence studies from other countries will add to our understanding of the global burden of SINV and the risk for potential SINV outbreaks.

## 1. Introduction

Sindbis virus (SINV) is a mosquito-borne alphavirus belonging to the *Togaviridae* family which circulates between mosquitoes and birds and only incidentally infects humans [1]. SINV was first isolated from *Culex* mosquitoes in 1952 in the Nile River delta in Egypt [2]. In humans, SINV infection can cause a febrile illness which may include arthralgia, rash, and malaise [1]. Chronic symptoms may last for months and even years following SINV infection and include musculoskeletal and other autoimmune disease-like symptoms [3,4]. Outbreaks of SINV disease have occurred primarily in Sweden (Ockelbo disease) [5], Finland (Pogosta disease) [6], Russia (Karelian fever) [7], South Africa [8], and Australia [9].

In Israel, antibodies against SINV were found in children and birds in the 1960s, and several SINV strains were isolated in 1967 and 1985 from a bird (*Streptopelia turtur*) and two mosquito species, *Culex perexiguus* and *Culex pipiens*, respectively [10,11,12]. Very recently, we found that 6.35% (191/3008 pools) of mosquito pools collected between 2004 and 2006 and 2013 and 2015 were positive for SINV RNA, suggesting that SINV is highly endemic and circulating in many populated areas in Israel [13]. Interestingly, SINV disease outbreaks have never been recorded in Israel and, moreover, SINV infection has rarely been diagnosed since testing began in 2004. This may suggest that: 1. The population in Israel is not infected with SINV; 2. SINV infection is asymptomatic; 3. There is low clinical alert for SINV among the medical community.

In order to examine whether SINV infects the Israeli population, we evaluated the seroprevalence of SINV IgG antibodies in samples obtained from persons representing all age groups in Israel and assessed correlates for being seropositive to SINV.

## 2. Materials and Methods

### 2.1. Study Design

A cross-sectional study was designed using serum samples from the National Sera Bank established by the Israel Center for Disease Control (ICDC) in 1997. The samples were residuals from diagnostic laboratories and healthy blood donors. Sera from subjects with confirmed or suspected immunological disorders were discarded. For all samples, basic demographic information was documented during the time of specimen collection, including patient age, gender, place of residence (city), birth country, and population group (“Jews and others” included Jews, non-Arabic Christians, and population not affiliated with a religion; “Arabs” included Muslims, Arab Christians, and Druze), as well as the date in which each sample was drawn. Socioeconomic status was established for each participant on the basis of his residential address using the socioeconomic residential classification and classified into low (socioeconomic class 1–5) and high (socioeconomic class 6–10).

### 2.2. Sampling

Israel was divided into six regions: South, Central, North (rural areas without metropolitan areas), Haifa (metropolitan area in the North), Jerusalem, and Tel-Aviv (two metropolitan areas in the Center), according to the Israel Central Bureau of Statistics database. A total of 3145 serum samples, collected between 2011 and 2014 from participants residing in these six regions, were included in the study (Table 1).

### 2.3. Antigen Preparation

SINV and mock antigen were prepared according to Simard et. al. [14] Briefly, the SINV antigen was prepared from Vero cells infected with a SINV strain isolated in Israel, while the mock antigen was obtained from non-infected Vero cells. Vero cell lysates were incubated overnight with polyethylene glycol (PEG) 6000 buffer and centrifuged at 4 °C at 10,000 *g* for 30 min. The pellets of SINV antigen or Mock were resuspended in PBS and kept in small aliquots at −70 °C.

### 2.4. Laboratory Testing

Indirect ELISA for detection of IgG antibodies was developed for this study using SINV-positive and -negative samples (a gift from Olli Vapalahti, University of Helsinki, Finland). Specifically, a 96-well microtiter Polysorb plate (Nunc, Thermo, Denmark) was coated overnight at 4 °C with optimal working concentrations of coating SINV antigen and mock antigen diluted 1:1000. After the plates were blocked with 5% skimmed milk at 37 °C for 30 min, human serum samples and controls (diluted 1:400 with 3% skimmed milk) were added to SINV antigen- and mock antigen-coated wells. The plates were incubated at 37 °C for 60 min and then washed, and goat anti-human IgG horseradish peroxidase (HRP)-conjugated antibodies (Jackson ImmunoResearch, PA, USA) (diluted 1:5000) were added to each well. Ortho-phenylenediamine (OPD), diluted in citric acid and hydrogen peroxide (H_2_O_2_), was used as a color substrate. After 15 min in the dark, the reaction was stopped by adding 150 µL stop solution (2 N sulfuric acid, H_2_SO_4_) to each well, and the optical density (OD) values were read at 450 nm. The ELISA index value for both test and control sera was determined by dividing the OD for each sample by its matching mock antigen. ELISA index values higher than 2.0, were considered positive.

### 2.5. SINV Neutralization Assay (SINV NT)

One hundred median tissue culture infectious dose (TCID_50_) of SINV (batch number M-514/02 isolated in Israel) was incubated with inactivated ELISA-positive sera diluted 1:10 to 1:1280 in 96-well plates for 60 min at 37 °C. Vero E-6 cells were added to each well and incubated for 4 days. Following Gentain violet staining (1%), which stained and fixed the cell culture layer, the neutralizing dilution of each serum sample was determined by identifying the well with the highest serum dilution without observable cytopathic effect. A dilution equal to 1:10 or above was considered neutralizing.

### 2.6. Data Analysis

Frequencies were calculated for the demographic characteristics distribution of the study population. Seropositivity rate was calculated by the number of positive samples divided by the number of samples tested. Logistic regression analyses were applied to assess the factors associated with seropositivity to Sindbis virus. The level of significance was determined at *p*-value of 0.05. Statistical analyses were performed using the SAS Enterprise Guide software package (version 7.12, SAS Institute Inc., Cary, NC, USA).

### 2.7. Ethics Statement

Sera collection was approved by the legal department of the Israeli Ministry of Health. All serum samples were collected anonymously and informed consent was not required.

## 3. Results

To evaluate the seroprevalence of SINV in the Israeli population, 3145 samples were tested for the presence of SINV IgG antibodies by ELISA. One hundred and thirteen samples were positive for SINV IgG by ELISA, which resulted in an overall seroprevalence of 3.6% (95%CI: 2.9–4.2%) (Table 1).

**Figure 1:**
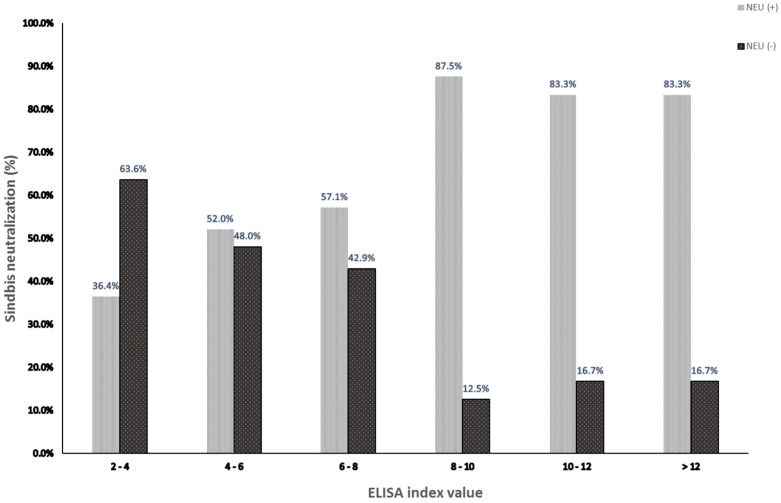
Neutralization of SINV ELISA IgG-positive samples. Serum neutralization assay was performed on 113 samples positive in the ELISA assay for SINV IgG. The samples were divided into six groups based on the ELISA index value. Percentages of samples neutralized by SINV [NEU (+)] or not [NEU (-)] are depicted.

To compare our ELISA IgG with the serological gold standard assay, SINV NT was performed on all 113 ELISA IgG-positive samples. Overall, 52.2% of the ELISA-positive samples neutralized a SINV strain isolated in Israel (Supplementary Table S1) resulting in an overall seroprevalence of 1.9% (95%CI: 1.4–2.4) (Table 1). To examine the correlation between a high ratio of positive/cut off in the ELISA assay (ELISA index value) and SINV NT, ELISA IgG-positive samples were divided into six groups based on their ELISA index value, and the percent of neutralizing samples (%neutralization) was assessed in each group. The results showed that 83.3–87.5% of samples with ELISA index value above 8 had neutralizing antibodies against SINV, while %neutralization of samples with ELISA index value below eight was only ~50% (Figure 1). Importantly, our ELISA showed 100% negative predictive value (NPV) and 100% sensitivity, since all 135 ELISA-negative samples (obtained from young adults) were also negative by SINV NT. This suggested that all ELISA-negative samples were true negatives, however, some false positive samples might be included in the ELISA-positive results. For this reason, both SINV NT and ELISA results were considered for the statistical calculations.

On the basis of the demographic characteristics of the study population, SINV seropositivity as tested by ELISA was significantly associated with older age (65–74 versus 0–4 years: OR = 3.71; 95%CI: 1.85–7.44), longer time living in Israel (≥60 versus 0–9 years; OR = 2.86; 95%CI 1.71–4.8), and residence area [South, Central, and North versus Jerusalem; OR = 2.67 (95%CI 1.13–6.32), OR = 3.73 (95%CI 1.41–9.86), OR = 2.66 (95%CI 1.12–6.32), respectively] (Table 2). Similarly, significant association of demographic characteristics with SINV NT seropositivity was demonstrated for age (30–54 and 65–74 versus 0–4 years: OR = 4.61; 95%CI: 1.32–16.17 and OR = 11.31; 95%CI: 3.28–8.98), longer time living in Israel (≥60 versus 0–9 years; OR = 4.34; 95%CI 2.01–9.36), residence type (urban versus rural; OR = 2.023; 95%CI 1.17–3.49), and residence area (North versus Jerusalem; OR = 2.58 95%CI 1.001–6.64) (Table 3). The multivariable analysis (Table 2 and Table 3) indicated that a high risk for being SINV IgG-seropositive was observed in people at the ages of 30–54 and 65–74 (OR = 5.56, 95%CI: 1.04–29.7 and OR = 6.51, 95%CI: 1.20–35.24, respectively, for ELISA and OR = 4.44, 95%CI: 1.27–15.58 and OR = 10.99, 95%CI: 3.18–37.91, respectively, for SINV NT). On the basis of the ELISA results, a higher risk for SINV IgG was observed for people residing in the south, central, and northern parts of the country excluding Tel-Aviv, Jerusalem, and Haifa metropolitan areas (OR = 3.07, 95%CI: 1.07–8.82, OR = 5.00, 95%CI: 1.58–15.80 and OR = 2.87, 95%CI: 1.04–8.23, respectively), while SINV NT showed a higher risk for SINV IgG in urban residence areas ( OR = 1.851, 95%CI: 1.06–3.22).

## 4. Discussion

The approach toward SINV characterization is somewhat peculiar. On the one hand, it is considered by the scientific community as the prototype for alpha virus and, as such, has been investigated thoroughly to study the underlying mechanism of human arbovirus infection using mouse models and cell culture [15,16]. On the other hand, because symptomatic SINV infections in humans are rare, mild, occur in several distinct areas around the world, and overall, are not considered a high burden on human health, very limited data have been collected about serological or clinical evidence in humans, despite endemic virus circulation in many countries in Europe, the Middle East, Africa, Asia, and Australia. We have recently found that the circulation of SINV in mosquitoes in Israel is very high and even exceeds the circulation of West Nile Virus (WNV), a flavivirus with a similar zoonotic cycle responsible for several outbreaks in the past years in Israel [13]. To gain a better understanding of the possible exposure of the Israeli population to SINV infection, we performed a large nationwide cross-sectional study and determined the seroprevalence of SINV antibodies among the Israeli population.

According to our study, the prevalence of IgG antibodies against SINV in the Israeli population in 2011–2014 was 3.6% as determined by ELISA assay and 1.9% as determined by SINV NT assay. The discrepancy between the results from ELISA and neutralization assays for arboviral diagnosis is not new and was already demonstrated by us and others for WNV [17,18,19,20]. Since our study measured SINV seroprevalence in the general population, it is much more likely that many of the SINV-positive persons were infected several years ago and therefore had lower levels of IgG which could be enough for detection by ELISA but not to neutralize the virus. Moreover, false-positive results due to ELISA cross reactivity was less likely, since no other alphavirus is known to circulate in our region. The true seroprevalence probably lies somewhere between 1.9 and 3.6%; nevertheless, because neutralization is considered the gold standard for serological diagnosis, we decided to calculate the association between demographic characteristics and SINV seropositivity according to both ELISA and SINV NT.

Up to now, seroprevalence of SINV was evaluated only in a few countries. An early study performed in Finland demonstrated an increase in SINV seroprevalence over the years, with 0.3% in the 1970s, 0.6% in the beginning of the 1980s, 1.4% after a SINV outbreak in 1981, and 1.8% at the end of the 1980s [6]. Two more recent studies evaluating the prevalence of SINV IgG antibodies in Finland during 1999–2003 found seroprevalence of 5.2% [21] and 11% [22], suggesting that SINV is becoming more prevalent in the Finnish population as the incidence of SINV disease increases. In Sweden, a significant difference in SINV seroprevalence was observed between different geographic zones. A seroepidemiological study from sera obtained between 1981 and 1987 found SINV seroprevalence of 3.6% in central Sweden, 0.1% and 1% in two regions in the north, and 1.8% and 0.2% in the south [23]. A 2009 population-based survey detected 2.9% SINV seroprevalence in northern Sweden, however, only adults over the age of 25 were tested [24]. Smaller studies in other countries also identified SINV antibodies in the general population: 1.5% in Iraq [25], 7.8% in Cameroon [26], and 2.6% in Kenya [27]. Altogether, our study highlights that there was no substantial difference in SINV seroprevalence between countries experiencing SINV outbreaks and countries which did not. It is therefore important to decipher the causes of SINV outbreaks and monitor SINV infection in the population of countries with known SINV circulation.

The multivariate analysis revealed an increase of SINV seroprevalence with age (Table 2 and Table 3). Several studies have previously similarly demonstrated that seroprevalence is higher with age, both for SINV [21] and for other vector-borne viral diseases such as WNV [17]. This observation fits very well with what we know about vector-borne viruses, i.e., the longer time a person is exposed, the higher the chances of encountering mosquitoes carrying SINV and as a result, the more frequent the infection with SINV. On the basis of the ELISA results, persons with IgG antibodies against SINV reside primarily outside Israel’s metropolitan areas (Table 2). Interestingly, the SINV NT results pointed to a higher prevalence in persons living in urban areas, but not in the metropolitan centers (Table 3). Indeed, most of the SINV-positive mosquitoes were collected in open areas in the south, center, and north regions of Israel [13]. Moreover, it is well documented that a higher circulation of *Culex* mosquitoes, SINV primary vector in Israel, is observed in the countryside and near ample water sources [28].

Most interesting is the difference we found within the same cohort of samples from the Israeli population between the seroprevalence of SINV (this study, 3.6% and 1.9%) and WNV (11.1%) [17]. Both SINV and WNV are highly abundant in Israeli *Culex* mosquitoes [13,29] and were found in 6.35% (from mosquito pools collected in 2003–2005 and 2013–2015) and 4.71% (from mosquito pools collected in 2000–2014) of mosquito pools analyzed, respectively. *Cx. perexiguus* and *Cx. pipiens*, which are both known to feed on mammals and birds [28], were infected with similar abundance by WNV and SINV. Therefore, our expectation was that WNV and SINV seroprevalence in the Israeli population would also be similar. A plausible explanation could be deduced from the significantly higher pathogenesis observed for WNV than for SINV, which may imply that natural antibodies are sometimes sufficient to protect against SINV infection and, as a result, no SINV-specific antibodies are generated [30,31]. Future studies should investigate this possibility both in vitro and in animal models.

## 5. Conclusions

In conclusion, after the identification of a high circulation of SINV in mosquitoes in many areas in Israel and in light of the lack of clinical data on SINV disease in Israel, we performed a nationwide cross-sectional study to investigate if Israeli SINV infects the human population. We demonstrated here that between 1.9 and 3.6% of the samples were positive for SINV IgG antibodies and that higher seroprevalence was observed at older age and outside metropolitan areas. Based on these results, future studies will try to uncover the differences in SINV infection between countries with SINV outbreaks, such as Finland and Sweden, and countries, such as Israel, with both circulating virus and human infection but no clinical presentations.

Because of the high mutation rates of these RNA viruses and former outbreaks of emerging viruses with no previously known pathogenesis, such as Zika virus, it is important to estimate the ability of circulating viruses to infect the population. Therefore, this study should interest many countries in Europe, the Middle East, Africa, and Australia with SINV circulation, as it demonstrates that SINV can infect the human population without apparent outbreaks or clinical presentations.

## Figures and Tables

**Table 1:** Demographic characteristics of the study population (*N* = 3,145). SINV NT: Sindbis virus Neutralization

		N Tested		ELISA			SINV NT		
				Seropositive		*p*-value*	Seropositive		*p*-value*
		N	%	N	%		N	%	
**Total**		3145	100	113	3.6		59	1.9	
Collection Year	2011	777	24.71	31	3.99	0.4632	18	2.32	0.7177
	2012	814	25.88	27	3.32		14	1.72	
	2013	785	24.96	33	4.2		15	1.91	
	2014	769	24.45	22	2.86		12	1.56	
Age Group (years)	0–4	460	14.63	13	2.83	<0.0001	3	0.65	<.0001
	5–9	426	13.55	12	2.82		7	1.64	
	10–14	432	13.74	10	2.31		3	0.69	
	15–29	509	16.18	8	1.57		6	1.18	
	30–54	476	15.14	19	3.99		14	2.94	
	55–64	388	12.34	15	3.87		6	1.55	
	65–74	246	7.82	24	9.76		17	6.91	
	75+	208	6.61	12	5.77		3	1.44	
Gender	Male	1623	51.61	54	3.33	0.4081	25	1.54	0.1520
	Female	1522	48.39	59	3.88		34	2.23	
Birth Country	Other	615	19.56	30	4.88	0.0565	15	2.44	0.2518
	Israel	2529	80.44	83	3.28		44	1.74	
Population group	Jews and Others	2591	82.52	92	3.55	0.7539	44	1.7	0.1050
	Arabs	549	17.48	21	3.83		15	2.73	
Residence Type	Rural	644	20.48	25	3.88	0.6586	20	3.11	0.0099
	Urban	2501	79.52	88	3.52		39	1.56	
Socioeconomic status	Low	1642	67.54	55	3.35	0.3802	26	1.58	0.9074
	High	789	32.46	32	4.06		12	1.52	
Residence Area	South	1095	34.82	44	4.02	0.0297	14	1.28	0.0021
	Central	253	8.04	14	5.53		5	1.98	
	North	1047	33.29	42	4.01		34	3.25	
	Haifa	193	6.14	2	1.04		0	0	
	Jerusalem	389	12.37	6	1.54		5	1.29	
	Tel-Aviv	168	5.34	5	2.98		1	0.6	
Number of years lived in Israel (years)	0–9	900	30.29	27	3.00	<0.0001	10	1.11	0.0001
	10–19	673	22.65	12	1.78		5	0.74	
	20–29	302	10.16	9	2.98		5	1.66	
	30–39	205	6.9	6	2.93		6	2.93	
	40–49	187	6.29	7	3.74		3	1.6	
	50–59	274	9.22	12	4.38		6	2.19	
	≥60	430	14.47	35	8.14		20	4.65	

* *p*-value represents significance of seropositivity between strata within each demographic characteristic.

**Table 2:** Univariate and multivariate logistic regression analyses of demographic characteristics of individuals diagnosed with Sindbis virus by ELISA.

		Univariate			Multivariate		
		OR	95%CI	*p*-value *	OR	95%CI	*p*-value
Collection Year	2011	Ref.					
	2012	0.83	0.49–1.40	0.4748			
	2013	1.06	0.64–1.74	0.8308			
	2014	0.71	0.41–1.24	0.2250			
Age Group (years)	0–4	Ref.					
	9–5	1.00	0.45–2.21	0.9934	0.95	0.43–2.12	0.9109
	10–14	0.81	0.35–1.88	0.6307	4.45	0.58–34.02	0.1503
	15–29	0.55	0.22–1.34	0.187	1.12	0.17–7.30	0.9018
	30–54	1.43	0.70–2.93	0.3289	5.56	1.04–29.71	0.0448
	55–64	1.38	0.65–2.94	0.4004	3.33	0.61–18.21	0.1648
	65–74	3.71	1.86–7.44	0.0002	6.51	1.20–35.24	0.0297
	75+	2.10	0.94–4.70	0.0690	3.85	0.69–21.39	0.1231
Gender	Male	0.85	0.59–1.24	0.4086			
	Female	Ref.					
Birth Country	Other	1.51	0.99–2.32	0.0581			
	Israel	Ref.					
Population group	Jews and Others	Ref.					
	Arabs	1.08	0.67–1.75	0.7539			
Residence Type	Rural	Ref.					
	Urban	1.11	0.70–1.74	0.6587			
Socioeconomic status	Low	0.82	0.53–1.28	0.3808			
	High	Ref.					
Residence Area	South	2.67	1.13–6.32	0.0252	3.08	1.07–8.82	0.0364
	Central	3.74	1.42–9.86	0.0077	5.00	1.59–15.79	0.0060
	North	2.67	1.12–6.33	0.0259	2.87	1.004–8.23	0.0492
	Haifa	0.67	0.13–3.34	0.6238	0.81	0.14–4.53	0.8075
	Jerusalem	Ref.					
	Tel-Aviv	1.96	0.59–6.51	0.2728	2.09	0.54–8.05	0.2859
Number of years lived in Israel (years)	0–9	Ref.					
	10–19	0.59	0.29–1.17	0.1288	0.17	0.03–1.15	0.0696
	20–29	0.99	0.46–2.14	0.9860	0.66	0.12–3.58	0.6293
	30–39	0.97	0.40–2.39	0.9557	0.20	0.04–1.18	0.0762
	40–49	1.26	0.54–2.93	0.5959	0.28	0.05–1.54	0.1424
	50–59	1.48	0.74–2.96	0.2674	0.39	0.08–1.98	0.2569
	≥60	2.86	1.71–4.80	<0.0001	0.65	0.13–3.19	0.5986

**Table 3:** Univariate and multivariate logistic regression analyses of demographic characteristics of individuals diagnosed with Sindbis virus by SINV NT.

		Univariate			Multivariate		
		OR	95%CI	*p*-value	OR	95%CI	*p*-value
Collection Year	2011	Ref.					
	2012	0.74	0.36–1.49	0.3980			
	2013	0.82	0.41–1.64	0.5772			
	2014	0.67	0.32–1.4	0.2841			
Age Group (years)	0–4	Ref.					
	5–9	2.54	0.65–9.91	0.1779	2.59	0.66–10.08	0.1704
	10–14	1.06	0.21–5.31	0.9385	1.09	0.22–5.43	0.9161
	15–29	1.82	0.45–7.31	0.4003	1.80	0.45–7.26	0.4062
	30–54	4.67	1.32–16.17	0.0168	4.44	1.27–15.58	0.0198
	55–64	2.39	0.59–9.63	0.2195	2.31	0.57–9.30	0.2396
	65–74	11.31	3.28–38.98	0.0001	10.98	3.18–37.91	0.0001
	75+	2.23	0.45–11.14	0.329	2.339	0.47–11.68	0.3022
Gender	Male	0.68	0.41–1.151	0.154			
	Female	Ref.					
Birth Country	Other	1.41	0.78–2.55	0.2540			
	Israel	Ref.					
Population group	Jews and Others	Ref.					
	Arabs	1.63	0.90–2.94	0.1083			
Residence Type	Rural	Ref.					
	Urban	2.02	1.17–3.49	0.0114	1.85	1.06–3.22	0.0294
Socioeconomic status	Low	1.04	0.52-2.07	0.908			
	High	Ref.					
Residence Area	South	0.99	0.36–2.78	0.9918			
	Central	1.55	0.44–5.40	0.4929			
	North	2.58	1.00–6.64	0.0498			
	Haifa	<0.001	<0.001–>999.999	0.9713			
	Jerusalem	Ref.					
	Tel-Aviv	0.46	0.05–3.97	0.4798			
Number of years lived in Israel (years)	0–9	Ref.					
	10–19	0.67	0.23–1.96	0.4603			
	20–29	1.50	0.51–4.42	0.4637			
	30–39	2.68	0.96–7.47	0.0588			
	40–49	1.45	0.39–5.32	0.5746			
	50–59	1.99	0.72–5.53	0.1858			
	≥60	4.34	2.01–9.36	0.0002			

## Supplementary Materials

The following are available online at https://www.mdpi.com/1999-4915/11/6/542/s1, Table S1: title, ELISA and SINV NT results.
