# Supplementary material for: Presence of Antibodies against Sindbis Virus in the Israeli Population: A Nationwide Cross-Sectional Study
Source: Viruses. 2019 Jun 11;11(6):542. doi: 10.3390/v11060542 (PMC6630228; doi:10.3390/v11060542)
Supplement: Supplementary file 1 [file viruses-11-00542-s001.pdf]

Supplementary Table 1: ELISA and SINV NT results

Sample No.	ELISA results (ELISA index value)	SINV NT (Cut off value)
1	Positive (3.56)	Negative
2	Positive (2.66)	Negative
3	Positive (4.76)	Negative
4	Positive (12.59)	Positive (1:10)
5	Positive (2.88)	Negative
6	Positive (3.09)	Negative
7	Positive (4.72)	Positive (1:10)
8	Positive (3)	Negative
9	Positive (2.36)	Positive (1:10)
10	Positive (3.53)	Positive (1:10)
11	Positive (4.02)	Positive (1:10)
12	Positive (10.08)	Positive (1:40)
13	Positive (2.44)	Negative
14	Positive (2.18)	Negative
15	Positive (4.67)	Negative
16	Positive (2.18)	Negative
17	Positive (2.43)	Negative
18	Positive (5.55)	Negative
19	Positive (4.48)	Positive (1:10)
20	Positive (4.16)	Negative
21	Positive (2.97)	Positive (1:20)
22	Positive (5.46)	Positive (1:20)
23	Positive (2.72)	Positive (1:20)
24	Positive (13.73)	Positive (1:40)
25	Positive (5.94)	Positive (1:20)
26	Positive (4.04)	Negative
27	Positive (3.04)	Negative
28	Positive (3.09)	Positive (1:20)
29	Positive (5.4)	Negative
30	Positive (6.08)	Positive (1:10)
31	Positive (3.49)	Negative
32	Positive (10.54)	Positive (1:10)
33	Positive (2.32)	Negative
34	Positive (2.83)	Negative
35	Positive (4.19)	Positive (1:10)
36	Positive (2.95)	Negative
37	Positive (2.5)	Negative
38	Positive (2.7)	Negative
39	Positive (2.32)	Negative
40	Positive (2.08)	Negative
41	Positive (3.53)	Negative
42	Positive (4.8)	Negative
43	Positive (5.59)	Positive (1:10)
44	Positive (2.92)	Negative
45	Positive (3.92)	Positive (1:10)
46	Positive (6.45)	Negative
47	Positive (3.15)	Negative

48	Positive (3.97)	Negative
49	Positive (3.91)	Positive (1:10)
50	Positive (6.32)	Positive (1:20)
51	Positive (3.81)	Positive (1:10)
52	Positive (6.48)	Negative
53	Positive (5.82)	Positive (1:20)
54	Positive (2.28)	Negative
55	Positive (2.74)	Negative
56	Positive (2.87)	Negative
57	Positive (2.35)	Negative
58	Positive (2.29)	Negative
59	Positive (6.73)	Positive (1:20)
60	Positive (5.29)	Negative
61	Positive (4.25)	Negative
62	Positive (2.3)	Negative
63	Positive (15.35)	Negative
64	Positive (3.86)	Positive (1:10)
65	Positive (2.52)	Negative
66	Positive (4.78)	Negative
67	Positive (8.22)	Positive (1:10)
68	Positive (7.51)	Negative
69	Positive (2.89)	Negative
70	Positive (5.78)	Negative
71	Positive (4.33)	Negative
72	Positive (2.19)	Negative
73	Positive (11.31)	Positive (1:10)
74	Positive (2.08)	Negative
75	Positive (8.9)	Positive (1:10)
76	Positive (3.62)	Positive (1:10)
77	Positive (5.25)	Positive (1:10)
78	Positive (3.44)	Positive (1:10)
79	Positive (12.66)	Positive (1:10)
80	Positive (12.7)	Positive (1:10)
81	Positive (12.99)	Positive (1:10)
82	Positive (11.67)	Positive (1:10)
83	Positive (2.33)	Positive (1:20)
84	Positive (2.62)	Negative
85	Positive (2.74)	Positive (1:20)
86	Positive (2.27)	Positive (1:10)
87	Positive (2.14)	Positive (1:10)
88	Positive (18.56)	Positive (1:10)
89	Positive (2.69)	Negative
90	Positive (10.31)	Negative
91	Positive (12.91)	Positive (1:10)
92	Positive (8.75)	Positive (1:10)
93	Positive (4.86)	Positive (1:10)
94	Positive (15.31)	Negative
95	Positive (8.31)	Negative
96	Positive (10.73)	Positive (1:10)
97	Positive (5.72)	Positive (1:20)
98	Positive (2.24)	Positive (1:10)
99	Positive (9.83)	Positive (1:10)

100	Positive (2.05)	Positive (1:10)
101	Positive (2.31)	Positive (1:40)
102	Positive (2.22)	Positive (1:40)
103	Positive (2.02)	Positive (1:20)
104	Positive (8.6)	Positive (1:10)
105	Positive (9.88)	Positive (1:10)
106	Positive (8.3)	Positive (1:10)
107	Positive (18.09)	Positive (1:10)
108	Positive (3.52)	Negative
109	Positive (24.18)	Positive (1:10)
110	Positive (12.94)	Positive (1:10)
111	Positive (7.89)	Positive (1:10)
112	Positive (4.89)	Positive (1:20)
113	Positive (4.59)	Positive (1:10)
